# Frataxin Deficiency Leads to Reduced Expression and Impaired Translocation of NF-E2-Related Factor (Nrf2) in Cultured Motor Neurons

**DOI:** 10.3390/ijms14047853

**Published:** 2013-04-10

**Authors:** Valentina D’Oria, Stefania Petrini, Lorena Travaglini, Chiara Priori, Emanuela Piermarini, Sara Petrillo, Barbara Carletti, Enrico Bertini, Fiorella Piemonte

**Affiliations:** 1 Confocal Microscopy Core Facility, Research Laboratories, Bambino Gesù Children’s Hospital, IRCCS, Rome 00165, Italy; E-Mails: vale_doria@yahoo.it (V.D.); stefania.petrini@opbg.net (S.P.); 2 Unit for Neuromuscular and Neurodegenerative Diseases, Bambino Gesù Children’s Hospital, IRCCS, Piazza S’Onofrio, 4, Rome 00165, Italy; E-Mails: lorena.travaglini@opbg.net (L.T.); chiara.priori@fastwebmail.it (C.P.); emanuela.piermarini@opbg.net (E.P.); sara.petrillo@opbg.net (S.P.); carletti.barbara@tiscali.it (B.C.); ebertini@gmail.com (E.B.)

**Keywords:** FRDA, frataxin, Nrf2, GSSG, oxidative stress, NSC34 neurons

## Abstract

Oxidative stress has been implicated in the pathogenesis of Friedreich’s Ataxia (FRDA), a neurodegenerative disease caused by the decreased expression of frataxin, a mitochondrial protein responsible of iron homeostasis. Under conditions of oxidative stress, the activation of the transcription factor NF-E2-related factor (Nrf2) triggers the antioxidant cellular response by inducing antioxidant response element (ARE) driven genes. Increasing evidence supports a role for the Nrf2-ARE pathway in neurodegenerative diseases. In this study, we analyzed the expression and the distribution of Nrf2 in silenced neurons for frataxin gene. Decreased Nrf2 mRNA content and a defective activation after treatment with pro-oxidants have been evidenced in frataxin-silenced neurons by RT-PCR and confocal microscopy. The loss of Nrf2 in FRDA may greatly enhance the cellular susceptibility to oxidative stress and make FRDA neurons more vulnerable to injury. Our findings may help to focus on this promising target, especially in its emerging role in the neuroprotective response.

## 1. Introduction

Oxidative stress has been implicated in the pathogenesis of Friedreich’s Ataxia (FRDA), an autosomal recessive disorder, neuropathologically characterized by prominent degeneration of the spinal cord pathways, with predominant neuronal loss in the dorsal root ganglia and in the Clarke’s columns, together with degeneration of their long tracts as well as involvement of the pyramidal tracts [1].

Most FRDA patients are homozygous for expanded GAA triplet repeat sequences (E alleles) in intron 1 of the *FXN* gene on chromosome 9q13 [2]. This mutation causes the formation of a “sticky” triplex DNA structure that interferes with correct transcription and reduces the synthesis of frataxin, a mitochondrial protein of 210 aminoacids, normally expressed at high levels in the human spinal cord [3,4]. The precise sequence of pathogenic events in FRDA remains uncertain. Current evidence suggests that the loss of frataxin impairs mitochondrial iron homeostasis resulting in respiratory chain enzymes dysfunction with excess in the free-radical production [5,6]. Reduced activities of mitochondrial enzymes containing iron–sulphur clusters as well as impairment of tissue energy metabolism have been demonstrated by biochemical and ^31^P-MRS studies in cardiac and skeletal muscle from FRDA patients [7–11]. A key role of oxidative stress in the pathophysiology of the disease has been supported by the finding of increased blood and urinary levels of oxidative stress markers in FRDA patients [12,13], as well as by *in vivo* evidence of the antioxidant enzymes impairment and imbalance of glutathione homeostasis [14,15].

Several model systems with frataxin deficiency exhibit oxidative stress [16]. Loss of aconitase activity, protein oxidation and high sensitivity to oxidants were found in frataxin depleted yeast cells [17–19] and in frataxin siRNA knockdown *Caenorhabditis elegans*[20]. In *Drosophila*, overexpression of frataxin increases the resistance to oxidative stress [21], whereas siRNA frataxin knockdown increases the susceptibility to iron toxicity and hydrogen peroxide [22,23]. Mouse conditional knockouts show increased ROS in pancreas and hepatocytes [24,25] and signs of oxidative stress have been found in the YG8 and YG22 mice expressing mutant human frataxin [26].

In this study, we silenced murine NSC34 neuroblastoma cells deriving from the fusion with spinal cord motor neurons for the gene of frataxin and evaluated the expression of the transcription factor Nrf2 that has a fundamental role in the antioxidant response to oxidative stress [27].

Nrf2 binds to antioxidant response element (ARE) and induces the expression of several detoxification enzymes, including heme oxygenase-1 [28], NAD(P)H:quinine oxidoreductase-1 [29,30], superoxide dismutase (SOD), glutathione *S*-transferases [31], as well as glutathione-synthesizing enzymes, glutamate-cysteine ligase catalytic subunit (GCLC) and glutamate-cysteine ligase modifier subunit (GCLM) [32–34].

To date, evidences on the expression of Nrf2 in FRDA, particularly focused on neuronal cells, are lacking. A defect in its expression has been recently found in dorsal root ganglia of YG8R mouse model of the disease [35] and impaired nuclear translocation has been evidenced in cultured dermal fibroblasts of FRDA patients [36].

Here, we investigated the expression and the intracellular distribution of Nrf2 in neuronal cells silenced for frataxin gene, in order to discover a possible role of Nrf2 in the pathogenesis of FRDA. We found a decrease in Nrf2 expression as a consequence of frataxin deficiency, thus suggesting a role for Nrf2 in the defective antioxidant response in FRDA, ultimately resulting in neurodegeneration. In light of these findings, we propose Nrf2 as promising target in the neuroprotection and its stimulation as a rational therapy in FRDA.

## 2. Results and Discussion

### 2.1. Frataxin Silencing in NSC34 Motor Neurons

**Figure 1:**
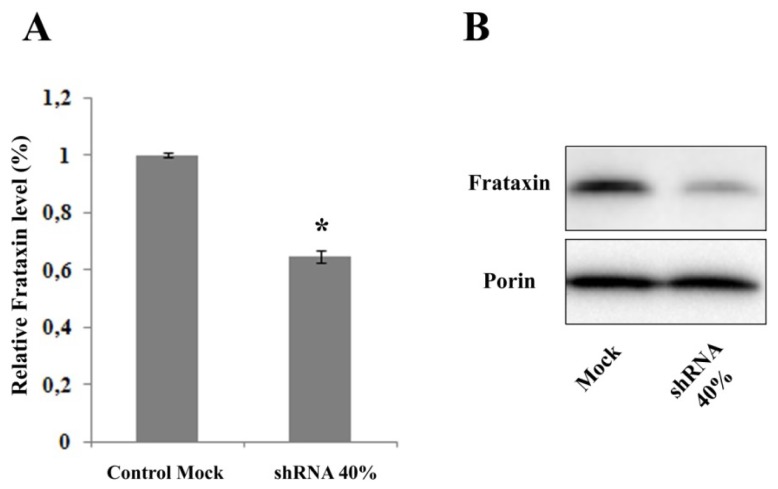
(**A**) RT-PCR frataxin level. NSC34 neurons were transduced with lentivectors encoding for shRNA sequences against murine frataxin (shRNA 40%) or containing only the *GFP reporter* gene (control Mock). The level of frataxin mRNA was quantified by real-time RT-PCR. The results were expressed as percentage of the control Mock-transfected cells. The error bars indicate SD (*p < 0.05); (**B**) Representative western blot of the frataxin protein level. Porin was used as reference protein.

Frataxin gene silencing has been carried out on NSC34 neurons deriving from the fusion of neuroblastoma cells with spinal cord motor neurons, by means of specific shRNA lentiviral vectors. By RT-PCR (Figure 1A), we obtained a 40% decrease of the frataxin mRNA (indicated as shRNA 40%), compared to the level of FXN mRNA in the control cell line consisting of cells infected with the GFP vector alone (reported as Mock).

### 2.2. Nrf2 Expression Is Decreased in Frataxin-Silenced Neurons

**Figure 2:**
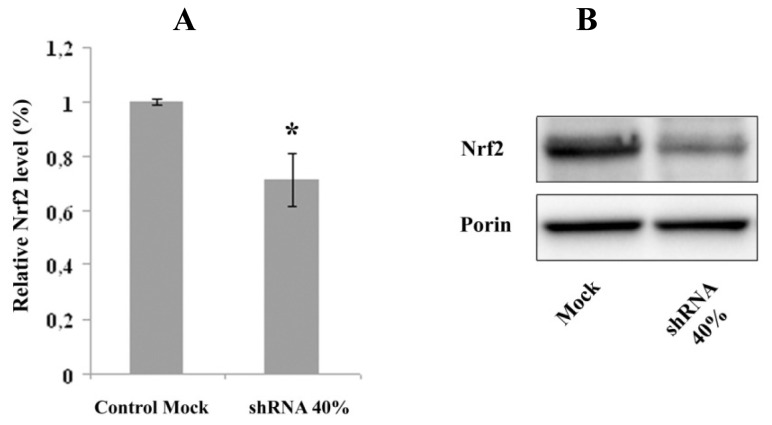
(**A**) RT-PCR NF-E2-related factor (Nrf2) level. Nrf2 mRNA transcripts were quantified in shRNA 40% and in control Mock neurons by real-time RT-PCR. The results were expressed as percentage of the control Mock transfected cells. The error bars indicate SD (*p < 0.05); (**B**) Representative western blot of the Nrf2 protein level. Porin was used as reference protein.

We examined transcript levels of Nrf2 in shRNA 40% by RT-PCR (Figure 2A) and western blot analysis (Figure 2B), and we found that neurons with reduced frataxin had about 30% decreased Nrf2 transcript, respect to control mock cells.

### 2.3. Decreased Expression of Nrf2-Targeted Genes in Frataxin-Silenced Neurons

**Figure 3:**
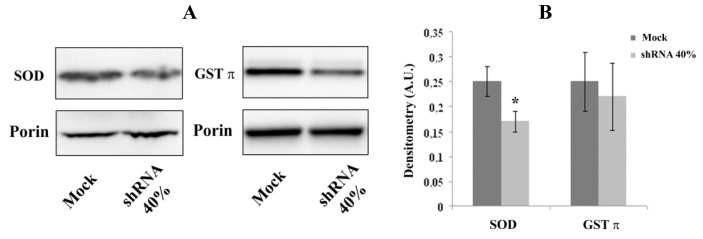
(**A**) Representative western blots of downstream Nrf2 target proteins. Forty microgrammes shRNA 40% and Mock neurons were applied onto 4%–12% Bis–Tris SDS-polyacrylamide gel electrophoresis as reported in Experimental Section, and probed with anti-SOD (1:1000 dilution) and anti-GST π (1:500 dilution) antibodies; (**B**) Densitometry of blots, normalized to porin. The error bars indicate SD (*p < 0.05). **A B**

According to the decreased Nrf2 transcript, by immunoblot analysis we evidenced a 30% reduction of the Nrf2-targeted gene protein SOD in shRNA 40% cells (Figure 3), thus supporting a defective antioxidant pathway in frataxin-depleted neurons. Also, glutathione *S*-transferase π GST π underwent a slight (12%) reduction.

### 2.4. Nrf2 Distribution in Frataxin-Silenced Neurons

**Figure 4:**
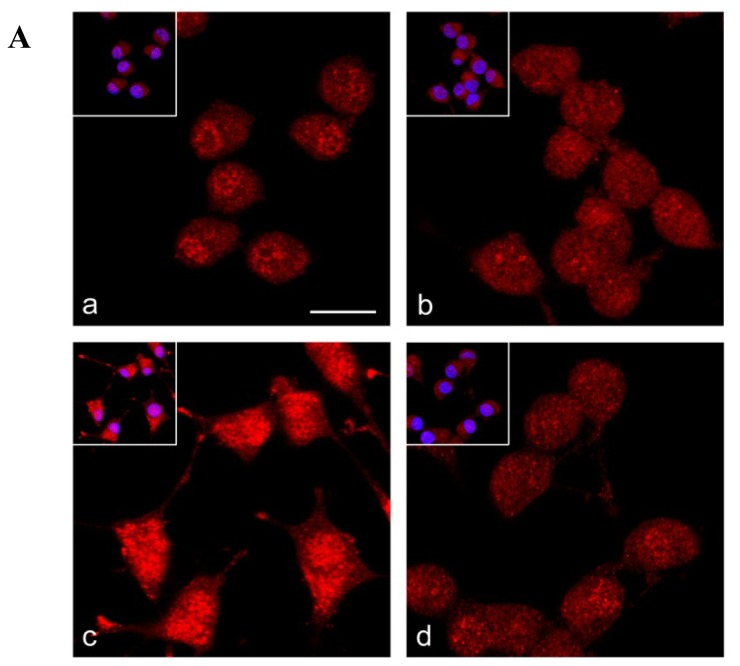

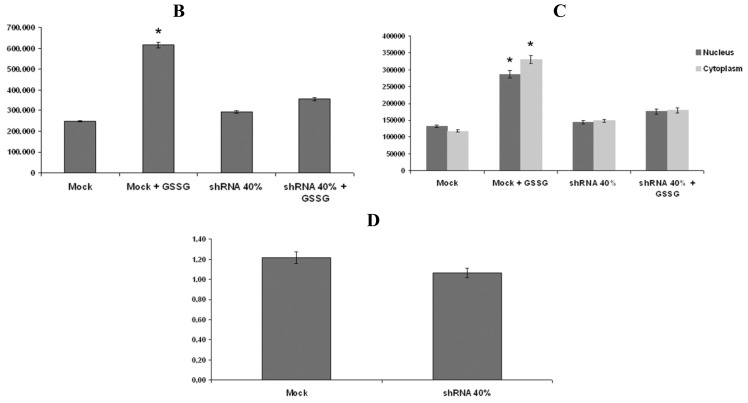
Nrf2 distribution in control (Mock) and shRNA 40% neurons under basal and oxidative stress conditions. (**A**) The confocal microscopy analysis of control (**a**, **c**) and frataxin-silenced (**b**, **d**) neurons immunolabelled with anti-Nrf2 antibody (*red*) revealed an increase of the Nrf2 signal in the nucleus respect to the cytoplasm in control Mock cells (**a**), whereas shRNA 40% neurons didn’t show any difference between the two compartments (**b**). After GSSG treatment, control Mock neurons (**c**) exhibited a significant increase of Nrf2 immunostaining in both nuclear and cytoplasmic areas, whereas frataxin-silenced cells did not respond to the stimulus (**d**). Nuclei were counterstained with Hoechst (*blue*, double staining in *insets*). Bar: 20 μm; (**B**–**C**) Total (**B**), nucleus/cytoplasm (**C**) Nrf2 fluorescence intensity in control Mock and shRNA 40% neurons before and after glutathione (GSSG) treatment; (**D**) Ratios of Nrf2 fluorescence intensities between nucleus and cytoplasm. Data are presented as the mean ± SEM (*p < 0.001). Nearly 150 nuclei were counted for each sample analyzed.

As the intracellular localization is critical for Nrf2 activation, we analyzed by immunofluorescence the distribution of Nrf2 in shRNA 40% and in mock neurons. As shown in Figure 4, the confocal imaging (panel A) and the fluorescence intensity analysis (panels B–D) revealed a different distribution of the Nrf2 signal between nucleus and cytoplasm in control mock neurons (Figure 4A, a) with a mild tendency to nuclear accumulation (mean ratio nucleus/cytoplasm: 1.22 ± 0.06; Figure 4D). In frataxin silenced shRNA 40% neurons, Nrf2 appeared equally distributed between nucleus and cytoplasm (Figure 4A, b), as also evidenced by the analysis of the ratio of Nrf2 fluorescence intensities between the two compartments (1.07 ± 0.05; Figure 4D).

### 2.5. Nrf2 Fails to Translocate to Frataxin Silenced Neurons Nuclei in Response to Oxidative Stress

The oxidized form of glutathione (GSSG) was used to induce oxidative stress. Upon exposure to a short GSSG pulse (20 min), Nrf2 showed a 3 times increase of total Nrf2 immunoreaction, both in the nucleus than in the cytoplasm, in control mock GSSG-treated neurons compared to untreated cells (total Nrf2 intensity 616.279 ± 94.888 *vs*. 249.021 ± 39.925; Figure 4A a and c, and Figure 4B–C). On the contrary, in frataxin-silenced shRNA 40% this Nrf2 activation following oxidative insult did not occur (total Nrf2 intensity 355.199 ± 70.044 *vs*. 292.522 ± 45.218; Figure 4A d, and B,C), supporting the defective Nrf2 response previously described in FRDA fibroblasts [36] and in dorsal root ganglia of a YG8R FRDA mouse model [35].

### 2.6. Discussion

Nrf2 is a transcription factor that regulates the expression of cytoprotective genes in response to oxidative stress [27,37].

Oxidative stress is associated with neuronal cell death in multiple chronic neurodegenerative diseases, including Alzheimer’s disease, Parkinson’s disease (PD), Huntington’s disease, and amyotrophic lateral sclerosis. Therefore, Nrf2-ARE activation may be a novel neuroprotective pathway that confers resistance to a variety of neurodegenerative insults.

Growing evidence support a role for the Nrf2 pathway in neurodegenerative diseases [27,38]. The expression of Nrf2 driven genes is high in the brain of patients with PD [39,40] and its activation protects against neurodegeneration in mouse models of familial amyotrophic lateral sclerosis and PD [41,42].

Recently, it has been demonstrated that frataxin expression is significantly correlated with Nrf2 expression in dorsal root ganglia of a FRDA mouse model [35]. Furthermore, defects in Nrf2 nuclear translocation [36] and in Nrf2-regulated genes [43] have also been reported in FRDA fibroblasts and in frataxin-depleted HeLa cells [35]. Thus, in light of these new implications of Nrf2 in the pathogenesis of FRDA, we decided to analyze Nrf2 expression in cultured motor neurons which were silenced for frataxin gene.

Our findings evidence lower levels of the Nrf2 transcript in frataxin depleted motor neurons and a defective Nrf2 activation after incubation with GSSG, the oxidized form of glutathione. The decrease of Nrf2 transcript reached about 30% in shRNA 40% compared to control neurons and this negatively affects protein levels of SOD and GST π, two of the downstream *Nrf2* gene targets. These results are consistent with those by Shan *et al.*[35] in DRG and cerebella of a FRDA mouse model, where frataxin-deficiency caused Nrf2 reduction at the transcript and protein targeted-genes level. In our model, a 40% frataxin decrease is enough to induce a defect in Nrf2 pathway, thus indicating a high sensitivity of cultured motor neurons to a mild silencing of frataxin gene expression. Nrf2 may represent an early sensor in the pathogenic cascade of the disease and require a lower threshold of silencing to be affected.

The neuronal loss of Nrf2 may greatly enhance the cellular susceptibility to oxidative stress and make FRDA neurons more vulnerable to injury. Paupe *et al.*[36] explain the defective Nrf2 response in FRDA fibroblasts as the result of an abnormal distribution of actin fibers to which, under basal conditions, is linked the Nrf2-Keap1 complex. Upon oxidation, Nrf2 dissociates from the microfilaments network and translocates into the nucleus, where it coordinates the activation of a battery of antioxidant genes expression [44]. It has been proposed that an abnormal distribution of actin microfilaments is responsible of the improper Nrf2 nuclear translocation observed in FRDA fibroblasts [36]. Microfilament abnormalities, due to actin oxidation, have been found in cultured dermal fibroblasts of patients with FRDA [45], thus supporting a contribution of cytoskeletal proteins to nucleus/cytoplasm Nrf2 distribution. However, the presence of a defective nuclear import-export in FRDA is still controversial, and no decreased Nrf2 transport to the nucleus has been evidenced in the frataxin-silenced HeLa and in DRG neurons of a mouse FRDA model [35].

The failure of Nrf2 activation observed in our frataxin-silenced motor neurons leads to a reduced expression of protein levels of SOD and this may further exacerbate the faulty antioxidant response.

As glutathione is the major antioxidant molecule in cells, and neurons are highly susceptible to redox changes of the GSH/GSSG ratio [46], we evaluated the antioxidant response of frataxin-silenced neurons by treatment with an excess of GSSG. Interestingly, in control neurons the Nrf2 immuoreactivity was consistently increased after GSSG exposure, whereas no Nrf2 activation was found in frataxin silenced neurons. This indicates that the defect of Nrf2 expression, resulting from frataxin deficiency, causes an inadequate response to antioxidants in FRDA, further worsening the pathological effects of the disease.

## 3. Experimental Section

### 3.1. Cell Culture

The NSC34 neuroblastoma cells, deriving from the fusion with spinal cord motor neurons (originally donated by Neil Cashman), were maintained in Dulbecco’s Modified Eagle Medium (DMEM, Invitrogen, Grand Island, NY, USA) supplemented with 10% fetal bovine serum (Invitrogen, Paisley, UK), 1% penicillin-streptomycin (20 U/mL, Invitrogen, Paisley, UK) and 1% glutamine (2 mM, Invitrogen, Paisley, UK). Cells were differentiated as described previously [47]. Briefly, cells were cultured at a density of 2000 cell/cm^2^ on tissue culture glass coverslips coated with 100 μg/mL of poly-d-lysine (Sigma, St. Louis, MO, USA). Cells were maintained for 6–7 days *in vitro* (d.i.v.) at 37 °C in a humidified atmosphere with 5% CO_2_, in DMEM/F12 (Invitrogen), supplemented with glutamine (2 mM; Invitrogen), penicillin-streptomycin (20 U/mL; Invitrogen), 1% fetal bovine serum (Invitrogen) and 1% non essential amminoacids (Sigma, St. Louis, MO, USA). For GSSG treatments, cells were incubated for 20 min, at 37 °C, with 100 μM (final concentration) GSSG methyl ester [Glu-(Gly-Cys-OMe)-OMe]^2^ (GSSGme, Peptide International, Louisville, KY, USA) diluted in cell culture medium.

### 3.2. Stable shRNA Cell Lines Generation

For infections, NSC34 cells were plated overnight at a density of 2 × 10^4^. ShRNA lentiviral particle encoding short-hairpin RNA sequences (Open Biosystem, Waltham, MA, USA), and hereafter reported as shRNA 40%, was diluted in maintaining medium without serum and antibiotics, and added to the cells for 5 h. Then, a further addition of complete medium was made to the cultures, and cells were collected after 72 h. Control cell lines consisted of cells infected with the GFP vector alone will be referred to as mock. Cell sorting was performed 96 h after infection. Trypsinized cells were resuspended in PBS and filtered through a 50 μm sterile mesh (BD Biosciences, NJ, USA) and sorted for GFP expression by using a FACSAria II cell sorter (BD Biosciences). Positive cells with the strongest GFP expression were collected and undergone to puromycin selection (10 μg/mL). Antibiotic selection was maintained for 7 days to obtain a stable cell line, which was finally analyzed for frataxin silencing.

### 3.3. Real-Time Quantitative RT-PCR

Total RNA was extracted with RNAzolB (Tel-Test Inc., Friendswood, TX, USA) according to the manufacturer’s instructions and quantified spectrophotometrically. cDNA synthesis was performed with 1 μg of DNase-treated RNA using ImProm-II reverse transcriptase (Promega, Madison, WI, USA) and oligodT as primer. Real-time PCR was carried out with PCR primers specific for frataxin mRNA (sense, 5′-gtggagatctaggaacctatg-3′, and antisense 5′-ttaaggctttagtgagctctg-3′) and a specific fluorogenic probe (5′-tccagtcataacgcttaggtccac-3′). The sense and antisense primers were designed to be complementary to regions on two different exons and the probe to span intron–exon boundaries to avoid amplification and recognition of genomic DNA. The mRNA encoding for the housekeeping gene glyceraldehyde-3-phosphate dehydrogenase (GAPDH) was used as an endogenous reference. The PCR reaction was carried out using the GeneAmp™ 5700 Sequence Detection System (Applied Biosystems, Foster City, CA). Results were normalized to GAPDH levels using the 2^−ΔΔCt^ method. A value of 1 was given to control samples chosen as calibrators and the sample values expressed the n-fold reduction of frataxin mRNA with respect to the calibrator (normalized dose = nd).

### 3.4. Western Blot Analysis of Frataxin and Nrf2 Protein Levels

Cell pellets were homogenized with cell lysis buffer (Cell Signaling, Danvers, MA, USA) with complete protease inhibitors and PSMF (Roche Applied Science, Penzberg, Germany). Forty microgrammes lysates were analyzed on 4%–12% Bis–Tris gels (Invitrogen). Electrophoresis was carried out according to the manufacturer’s recommendations. After electrophoresis, proteins were transferred to nitrocellulose membranes by the iBlot device (Invitrogen), blocked with Odyssey blocking buffer (LI-COR Biotechnology, Lincoln, NE, USA) for 1 h, and incubated overnight with the primary anti-Frataxin antibody (1:250, Millipore, Temecula, CA, USA) and anti Nrf2 (AbC20, dilution 1:500, Santa Cruz Biotechnology, Santa Cruz, CA, USA). An antibody against porin (Mitosciences, Abcam, UK) was used as the reference protein (1:10,000 dilution).

### 3.5. Western Blot Analysis of Nrf2 and Downstream Target Proteins

Cell pellets were homogenized and processed as above, and incubated overnight with the primary anti-SOD antibody (1:1000, Stressgen/Enzo, Farmingdale, NY, USA) and anti GST π (1:500, Novocastra Laboratories, UK).

### 3.6. Immunocytochemistry

Adherent cells on coverslips were fixed with ice-cold 4% formaldehyde in phosphate buffered saline (PBS, pH 7.4) for 10 min, washed with PBS, permeabilized in 0.15% Triton X-100, and treated with 5% bovine albumin serum (BSA, 30 min). Then, samples were incubated overnight with polyclonal rabbit anti-Nrf2 (1:25, Santa Cruz Biotechnology) and revealed with anti-rabbit IgG conjugated to Alexa Fluor 555 (Invitrogen/Molecular Probes, Carlsbad, CA, USA). Glass cover slips were mounted on microscope slides with Tris–glycerol supplemented with 10% Mowiol (Calbiochem, La Jolla, CA, USA) to reduce fading of fluorescence. Negative controls were performed using 1% PBS/BSA without the primary antibody. Nuclear staining was performed with Hoechst 33342 (Invitrogen, Paisley, UK). All experiments were repeated thrice.

### 3.7. Imaging Analysis

The confocal microscopy imaging was performed on Olympus Fluoview FV1000 confocal microscope equipped with FV10-ASW version 2.0 software, using 60× (1.42 numerical aperture) oil objective. Optical single sections were acquired with a scanning mode format of 1024 × 1024 pixels, sampling speed of 40 μs/pixel, and 12 bits/pixel images. Fluorochromes unmixing was performed by acquisition of automated-sequential collection of multi-channel images, in order to reduce spectral crosstalk between channels. The intensity average of Nrf2 fluorescence was calculated using MetaMorph software, from cytometric measurements relative to total cell area as well as nuclear and cytoplasmic compartments, in six digital images randomly selected and analyzed for each immunostained cellular sample.

### 3.8. Statistical Analysis

Statistical differences were calculated using the Student’s *t*-test and data are presented as mean ± standard deviation (SD) or standard error of the mean (SEM). *p* < 0.05 was set as significant.

## 4. Conclusions

Nrf2 plays an important role in protecting cells from toxic insult. Our study highlights, for the first time, defects in Nrf2 expression and nuclear translocation in FRDA, a mitochondrial neurodegenerative disease increasingly considered “a redox disease”. Given the crucial role of Nrf2 in the antioxidant cellular response, our findings may help to focus on this promising target of the neuroprotective response.

## Abbreviations

FRDA: Friedreich’s Ataxia
Nrf2: NF-E2-related factor
ARE: antioxidant response element
GSH: reduced glutathione
GSSG: oxidized glutathione
